# Three novel obese indicators perform better in monitoring management of metabolic syndrome in type 2 diabetes

**DOI:** 10.1038/s41598-017-10446-3

**Published:** 2017-08-29

**Authors:** Chun-Ming Ma, Na Lu, Rui Wang, Xiao-Li Liu, Qiang Lu, Fu-Zai Yin

**Affiliations:** Department of Endocrinology, The First Hospital of Qinhuangdao, No.258 Wenhua Road, Qinhuangdao, 066000 Hebei Province China

## Abstract

The present study evaluated the performance of three novel obese indicators, visceral adiposity index (VAI), lipid accumulation product (LAP) and waist circumference-triglyceride index (WTI), for identifying metabolic syndrome(MetS) in type 2 diabetes. A cross-sectional study was conducted on 711 type 2 diabetes in Qinhuangdao. The MetS was defined as the definition of Chinese Diabetes Society. Receiver operating characteristic curve analyses were performed to assess the accuracy of three obese indicators as diagnostic tests for MetS. The prevalence of MetS was 71.3%. In men, among all three obese indicators, the LAP had the highest area under curve (AUC) value (AUC = 0.894), followed by VAI (AUC = 0.860) and WTI (AUC = 0.855). In women, among all three obese indicators, the LAP had the highest AUC value (AUC = 0.906), followed by WTI (AUC = 0.887) and VAI (AUC = 0.881). However. there was no significant difference between the three obese indicators(*P* > 0.05). Three obese indicators were effective indicators for the screening of MetS, LAP and WTI are more simple.

## Introduction

Diabetes mellitus is one of the most important public health challenges of the twenty-first century^1^. China is one of the fastest developing countries in the world. Rapid economic progress has resulted in changes to both diet and physical activity. In China, the prevalences of diabetes and prediabetes reached 9.7% and 15.5%, respectively^2^. A close link exists between diabetes, cardiovascular disease and stroke, which are the most prevalent causes of morbidity and mortality in diabetic patients^3, 4^.

Metabolic syndrome (MetS) is a cluster of metabolic abnormalities, characterized as central obesity, elevated blood pressure, dysglycemia, elevated triglyceride (TG) levels, and low high-density lipoprotein cholesterol (HDL-C) levels. The prevalence of MetS is high in type 2 diabetes^5, 6^. MetS increases the risk of atherosclerotic vascular disease in type 2 diabetes^7^. In two prospective studies, MetS at baseline is associated with an increased risk of incident cardiovascular disease^8, 9^. MetS also increases the risk of stroke and is associated with stroke recurrence in type 2 diabetes^10, 11^.

Early identification and treatment of individuals with MetS could reduce risk of developing relevant diseases. Visceral adiposity index (VAI) and lipid accumulation product (LAP) are two novel indexes used for identifying MetS. Recently, several studies supported the use of these indexes for the screening of MetS in healthy population^12–15^, elderly^16, 17^, polycystic ovary syndrome^18–20^, and adult growth hormone deficiency^21^. Waist circumference-triglyceride index (WTI) is a predictor for the development of coronary artery disease^22^. To our knowledge no investigators have attempted to use these obese indicators for screening MetS in type 2 diabetes. The aim of our study was to evaluate the performance of three obese indicators, VAI, LAP and WTI, for identifying MetS in type 2 diabetes.

## Methods

### Subjects

After obtaining informed consent from subjects with type 2 diabetes a cross-sectional study was conducted. All subjects were recruited from the First Hospital of Qinhuangdao. All subjects were men and women over 18 years of age, with a diagnosis of type 2 diabetes (based on American Diabetes Association diagnostic criteria)^23^. The exclusion criteria included the following: (1) subjects with type 1 diabetes, (2) subjects with clinical evidence of other endocrinopathy, such as Cushing’s syndrome, hyperthyroidism, *et al*. (3) subjects were taking medications known to affect lipid metabolism, such as glucocorticoids, fibrates and statins, (4) subjects with acute and chronic inflammation. This study was approved by the ethics committee of the First Hospital of Qinhuangdao. All subjects provided written informed consent before study initiation. All experiments were performed in accordance with relevant guidelines and regulations.

### Measurements

Anthropometric measurements, including height, weight and waist circumference(WC) were obtained while the subjects were in light clothing and barefoot. Height and weight were measured to the nearest 0.1 cm and 0.1 kg, respectively. WC was accurately measured at the level of midway between the lowest rib and the top of the iliac crest. All measurements were taken twice, and the two measurements were averaged for analysis. Blood pressure was measured three times with a mercury sphygmomanometer while the subjects were seated after 10 min of rest, and the three measurements were averaged for analysis. Body mass index(BMI) was calculated by dividing weight (kg) by height squared (m^2^). Waist-to-height ratio(WHtR) was calculated by dividing the WC by height.

After a 10-hour overnight fast, blood samples were collected from an antecubital vein into heparinised tubes. Fasting plasma glucose(FPG) concentration was measured using the glucose oxidase method, and serum lipid levels were measured using enzymatic assays with an autoanalyzer (Hitachi, Tokyo, Japan).

VAI, a sex-specific index based on WC, BMI, TG and HDL-C, was calculated as follows^24^: Males: VAI = (WC/(39.68 + (1.88 × BMI))) × (TG/1.03) × (1.31/HDL), Females: VAI = (WC/(36.58 + (1.89 × BMI))) × (TG/0.81) × (1.52/HDL). LAP was calculated as (WC (cm) − 65) × TG (mmol/L) for men, and (WC (cm) − 58) × TG (mmol/L) for women^25^. We used the following formula to calculate the WTI = WC(cm) × TG(mmol/L)^22^.

### Definition of metabolic syndrome

Metabolic syndrome was defined using the definition of Chinese Diabetes Society. Participants had to meet any 3 or more of the following 5 factors: (1) WC ≥90 cm(male) and 85 cm(female), (2) FPG ≥6.1 mmol/L or 2-h plasma glucose levels ≥7.8 mmol/L after a 75-g oral glucose tolerance test (OGTT) or have been diagnosed with diabetes, (3) blood pressure ≥130/85 mmHg or have been diagnosed with hypertension, (4) TG ≥1.7 mmol/L, 5) HDL-C < 1.04 mmol/L^26^.

### Statistical Analyses

All analyses were performed using the SPSS 11.5 statistical software (SPSS 11.5 for Windows; SPSS, Inc., Chicago, IL). Numerical variables were reported as mean ± standard deviation. Comparisons were conducted between groups using the *t* test. Comparison of prevalence data was performed by *χ* ^2^ analysis. For each obese indicators, we divided them into increasing sex-specific quartile values and used logistic regression analysis to calculate odds ratios (ORs) and 95% confidence intervals (CIs) for MetS across quartiles, with quartile 1 as reference group, adjusting for potentially confounding variables such as age, BMI, drink, smoke and history of diabetes. By using receiver operating characteristic (ROC) analysis, ROC curves of each obese indicators were drawn to show how well they could separate subjects into groups with or without MetS. A test with an area under curve (AUC) of ≥0.85 is considered an accurate test^27^. Sensitivity and specificity of each obese indicators have been calculated at all possible cutoff points to find the optimal cutoff values. The optimal sensitivity and specificity were the values yielding maximum sums from the ROC curves. The comparisons of AUCs were performed with MedCalc 11.4.2.0 software (Ostend, Belgium). *P* < 0.05 was considered statistically significant.

## Results

Among these subjects, 71.3% were characterized by MetS. The prevalence of MetS was similar between men and women (men 72.1% vs women 70.3%, *χ* ^2^ = 0.261, *P* = 0.610). Characteristics of the study population are presented in Table 1. The age, history of diabetes, drink, smoke and FPG between the two groups were similar (*P* > 0.05). The levels of BMI, WC, WHtR, SBP(systolic blood pressure), DBP(diastolic blood pressure), TG, VAI, LAP and WTI were all significantly higher in MetS group than in non-MetS group (*P* < 0.001). The level of HDL-C was significantly lower in MetS group than in non-MetS group (*P* < 0.001).

**Table 1:** Basic characteristics of the study population.

Variables	Non-MetS group (n = 204)	MetS group (n = 507)	*t* or *χ* ^2^	*P*
Men/Women	112/92	289/218	0.261	0.610
Age (year)	54.4 ± 12.9	54.1 ± 12.8	0.291	0.771
History of diabetes (year)	6.8 ± 7.1	6.4 ± 6.7	0.681	0.496
Drink[n (%)]	67(32.8)	136(26.8)	2.583	0.108
Smoke[n (%)]	66(32.4)	153(30.2)	0.323	0.570
BMI (kg/m^2^)	23.9 ± 3.5	27.2 ± 3.6	10.869	0.000
WC (cm)	84.8 ± 8.7	95.8 ± 10.2	14.320	0.000
WHtR	0.51 ± 0.05	0.57 ± 0.06	12.473	0.000
SBP (mmHg)	121.6 ± 14.2	131.7 ± 18.3	7.864	0.000
DBP (mmHg)	78.4 ± 8.6	83.2 ± 9.8	6.490	0.000
FPG (mmol/L)	8.8 ± 4.3	9.1 ± 3.5	0.720	0.473
TG (mmol/L)	1.17 ± 0.57	2.96 ± 2.81	13.605	0.000
HDL-C (mmol/L)	1.29 ± 0.28	1.04 ± 0.29	10.350	0.000
VAI	1.48 ± 0.78	5.33 ± 6.28	13.523	0.000
LAP	26.9 ± 15.4	101.0 ± 109.2	14.910	0.000
WTI	99.8 ± 47.5	285.1 ± 282.2	14.286	0.000

MetS: metabolic syndrome; BMI: body mass index; WC: waist circumference; WHtR: waist to height ratio; SBP:systolic blood pressure; DBP:diastolic blood pressure; FPG: fasting plasma glucose; TG: triglyceride; HDL-C: high-density lipoprotein cholesterol; VAI: visceral adiposity index; LAP: lipid accumulation product; WTI: waist circumference-triglyceride index.

In men, among all three obese indicators, the LAP had the highest AUC value (AUC = 0.894), followed by VAI (AUC = 0.860) and WTI (AUC = 0.855). However. there was no significant difference between the three obese indicators(*P* > 0.05). LAP had the Youden’s index of 0.65 (Sen = 83.0%, Spe = 82.1%), with the optimal cut-off as 35.7. VAI had the Youden’s index of 0.60 (Sen = 71.9%, Spe = 88.3%), with the optimal cut-off as 2.00. WTI had the Youden’s index of 0.63 (Sen = 72.6%, Spe = 91.0%), with the optimal cut-off as 148.1. The AUC of LAP was higher than the AUCs of WC and WHtR (*P* < 0.05). The AUCs were similar between VAI, LAP and WTI in men (*P* > 0.05) (Table 2).

**Table 2:** The cut-off, sensitivities, specificities, Youden’s index and area under curve of each variable for the screening of metabolic syndrome in men and women.

Variables	Cut-off	Sensitivity	Specificity	Youden’s index	AUC (95%CI)	*P*	P*	*P* ^*#*^
**Men**								
WC	90.0	85.4	75.8	0.61	0.834 (0.789~0.878)	0.000		
WHtR	0.53	77.8	78.5	0.56	0.819 (0.773~0.865)	0.000	0.644	
VAI	2.00	71.9	88.3	0.60	0.860 (0.824~0.897)	0.000	0.383	0.169
LAP	35.7	83.0	82.1	0.65	0.894 (0.863~0.925)	0.000	0.032	0.007
WTI	148.1	72.6	91.0	0.63	0.855 (0.817~0.893)	0.000	0.490	0.237
**Women**								
WC	85.0	85.3	68.4	0.53	0.786 (0.725~0.847)	0.000		
WHtR	0.54	77.0	68.4	0.45	0.765 (0.703~0.827)	0.000	0.637	
VAI	2.28	78.4	83.6	0.62	0.881 (0.844~0.918)	0.000	0.009	0.001
LAP	44.0	84.4	80.4	0.65	0.906 (0.874~0.939)	0.000	0.000	0.000
WTI	151.7	70.6	93.4	0.64	0.887 (0.852~0.922)	0.000	0.004	0.000

AUC: area under curve; CI: confidence interval; WC: waist circumference; WHtR: waist to height ratio; VAI: visceral adiposity index; LAP: lipid accumulation product; WTI: waist circumference-triglyceride index. *Compared with WC, ^#^Compared with WHtR. The AUCs were similar between VAI, LAP and WTI(*P* > 0.05).

In women, among all three obese indicators, the LAP had the highest AUC value (AUC = 0.906), followed by WTI (AUC = 0.887) and VAI (AUC = 0.881). However. there was no significant difference between the three obese indicators(*P* > 0.05). LAP had the Youden’s index of 0.65 (Sen = 84.4%, Spe = 80.4%), with the optimal cut-off as 44.0. WTI had the Youden’s index of 0.64 (Sen = 70.6%, Spe = 93.4%), with the optimal cut-off as 151.7. VAI had the Youden’s index of 0.62 (Sen = 78.4%, Spe = 83.6%), with the optimal cut-off as 2.28. The AUCs of VAI, LAP and WTI were higher than the AUCs of WC and WHtR (*P* < 0.01). The AUCs were similar between VAI, LAP and WTI in women (*P* > 0.05) (Table 2).

The VAI, LAP and WTI were strongly associated with the odds of having MetS in both men and women, after adjustment for age, BMI, drink, smoke and history of diabetes. ORs for MetS increased with increasing quartiles of all variables(*P* < 0.001) (Tables 3, 4 and 5).

**Table 3:** Prevalence of metabolic syndrome according to the level of visceral adiposity index.

Men (n = 401)	*n* (%)	OR (95%CI)	*P*	Women (n = 310)	*n* (%)	OR (95%CI)	*P*
Quartile 1 (n = 100)	36 (36.0)	1		Quartile 1 (n = 77)	23 (29.9)	1	
Quartile 2 (n = 100)	61 (61.0)	2.79 (1.46~5.32)	0.002	Quartile 2 (n = 77)	48(62.3)	3.12 (1.50~6.49)	0.002
Quartile 3 (n = 100)	91 (91.0)	21.23 (8.61~52.32)	0.000	Quartile 3 (n = 78)	69(88.5)	13.24 (5.40~32.44)	0.000
Quartile 4 (n = 101)	101 (100.0)	160.05 (20.71~1236.39)	0.000	Quartile 4 (n = 78)	78 (100)	149.99 (19.30~1165.27)	0.000

Multiple logistic regression analysis, adjustment for age, body mass index, drink, smoke and history of diabetes.

**Table 4:** Prevalence of metabolic syndrome according to the level of lipid accumulation product.

Men (n = 401)	*n* (%)	OR (95%CI)	*P*	Women (n = 310)	*n* (%)	OR (95%CI)	*P*
Quartile 1 (n = 100)	29 (29.0)	1		Quartile 1 (n = 77)	19 (24.7)	1	
Quartile 2 (n = 100)	65 (65.0)	3.59 (1.82~7.07)	0.000	Quartile 2 (n = 77)	50 (64.9)	4.56 (2.19~9.51)	0.000
Quartile 3 (n = 100)	94 (94.0)	30.77 (11.58~81.79)	0.000	Quartile 3 (n = 78)	71 (91.0)	22.32 (8.27~60.23)	0.000
Quartile 4 (n = 101)	101(100.0)	159.75 (20.56~1241.07)	0.000	Quartile 4 (n = 78)	78 (100.0)	162.99 (20.53~1293.53)	0.000

Multiple logistic regression analysis, adjustment for age, body mass index, drink, smoke and history of diabetes.

**Table 5:** Prevalence of metabolic syndrome according to the level of waist circumference-triglyceride index.

Men (n = 401)	*n* (%)	OR (95%CI)	*P*	Women (n = 310)	*n* (%)	OR (95%CI)	*P*
Quartile 1 (n = 100)	36 (36.0)	1		Quartile 1 (n = 77)	24 (31.2)	1	
Quartile 2 (n = 100)	61 (61.0)	2.28 (1.19~4.35)	0.013	Quartile 2 (n = 77)	44 57.1)	1.89 (0.91~3.92)	0.084
Quartile 3 (n = 100)	94 (94.0)	25.78 (9.58~69.31)	0.000	Quartile 3 (n = 78)	72 (92.3)	16.47 (6.05~44.88)	0.000
Quartile 4 (n = 101)	98 (97.0)	35.80 (11.34~112.98)	0.000	Quartile 4 (n = 78)	78 (100.0)	108.31 (13.99~838.15)	0.000

Multiple logistic regression analysis, adjustment for age, body mass index, drink, smoke and history of diabetes.

## Discussion

In this population, we found that 71.3% of participants had MetS. The prevalence of MetS was very common in type 2 diabetes, which is similar to previous study^5, 6^. The performances of WC and WHtR were similar in our study. Several visceral adiposity indicators, such as LAP in men and LAP, WTI, VAI in women appeared to be better indices to predict presence of MetS, relative to WC and WHtR. Our study presents evidence that the LAP may simple and useful tool for the screening of MetS in type 2 diabetes.

It is now widely acknowledged that, the core of MetS is central obesity (also known as visceral adiposity), overweight with adipose tissue accumulation particularly around the waist. The evaluate of visceral adipose tissue, such as magnetic resonance imaging and computed tomography, is very expensive, may involve exposure to radiation, and their availability is limited. WC is the most accurate surrogate marker of visceral adiposity in adults, and are good indicators of insulin resistance^28, 29^. However, two pepole are the same in WC but different in height, so the cardiovascular risk factors maybe different. WHtR is defined as their WC divided by their height and has been widely recommended^30^. But the performances of WC and WHtR were similar in our study. Robust statistical evidence from studies involving 12 studies(men) and 13 studies(women) in several ethnic groups, shows the improvement in AUC (WHtR > WC) for detecting MetS is slightly in men (0.003) and women(0.009)^31^.

WC may be not appropriate for identifying MetS on its own. First, metabolically obese, normal-weight (MONW) individuals are very common in type 2 diabetes^32^. Second, obesity is a heterogeneous condition, thus metabolic abnormalities and cardiometabolic risk vary among obese individuals, with a significant proportion considered to be “metabolically healthy”^33^. Kahn *et al*. developed the LAP as an accurate index for identifying United States adults at cardiovascular risk^25^. LAP is an index based on a combination of WC and TG. In our study, LAP is preferred to WC and WHtR, both men and women. Dual metabolic defects are required to produce hypertriglyceridemia in obese subjects with similar levels of visceral adiposity. Mirmiran *et al*. found that LAP is associated with insulin resistance, lipid peroxidation, and systemic inflammation in type 2 diabetic patients^34^. Sambataro *et al*. found that LAP in relationships with insulin sensitivity (evaluated by the quantitative insulin sensitivity check index) in overweight type 2 diabetic subjects^35^. These results emphasize the clinical importance of assessing LAP in type 2 diabetes to identify subjects at high cardiovascular risk. WTI is similar to LAP^22^. Compared with LAP, WTI has better specificities but poor sensitivities.

VAI is an index estimated with the use of both anthropometric (BMI and WC) and metabolic (TG and HDL-C) parameters. This index is more complicated, but is not superior to LAP and WTI. It is consistent with previous results. In elderly, the VAI showed the lowest discriminatory power for MetS^16^. In the Chinese rural adults, the AUC of VAI for the screening of MetS was less than that of LAP in both men and women^36^.

There were differences between the results of VAI and WTI in men and women. The AUCs of WC and WHtR in women were slightly lower than men. The AUCs of LAP and WTI in women were slightly higher than men. Therefore, LAP and WTI appeared to be better indices to predict presence of MetS only in women, relative to WC and WHtR. The results suggest that WC may be more practical in men. Kim *et al*. found that the diagnostic values of the visceral fat area and WC for predicting the presence of multiple metabolic risk factors are influenced by gender. Although the WC and visceral fat area are similar in men, the WC is inferior to the visceral fat area in women^37^. WC correlates with hepatic fat accumulation in male Japanese patients with non-alcoholic fatty liver disease, but not in females^38^.

There are limitations to our study. First, the LAP was derived from studies of white, non-Hispanic blacks and Mexican Americans and the VAI was established in Caucasian populations^24, 25^. The suitability for other populations need to be further investigated. Second, the relationship between LAP, VAI, WTI and diabetic complication should be studied in the futrure. Third, the study population was relatively small. A larger sample size may be helpful in further strengthen the conclusion. Fourth, the levels of insulin were not measured in our study. We can not compare the performances of LAP, VAI and insulin resistance. Fifth, I did not record the information of excluded patients. This maybe cause some selective bias.

In summary, the prevalence of MetS was high in type 2 diabetes. Three novel obese indicators were effective indicators for the screening of MetS, LAP and WTI are more simple.
